# 4. A challenging case of refractory BehÇet's disease in an adolescent with sight threatening uveitis

**DOI:** 10.1093/rap/rky031.003

**Published:** 2018-09-20

**Authors:** Gisella Cooper, Jessica Choi, Anne-Marie McMahon, Clare Nash, Lisa Dunkley, Rachel Tattersall, Fahd Quhill, Helen Lee, Jenny Edgerton, F Welch, Ruud Verstegen, Daniel Hawley

**Affiliations:** 1 Paediatric Rheumatology, Sheffield Children's Hospital, Sheffield, UNITED KINGDOM; 2 Paediatric Ophthalmology, Sheffield Children's Hospital, Sheffield, UNITED KINGDOM; 3 Adult and Adoloescent Rheumatology, Sheffield Teaching Hospitals, Sheffield, UNITED KINGDOM; 4 Adolescent and Paediatric Rheumatology, Sheffield Children's Hospital, Sheffield, UNITED KINGDOM; 5 Rheumatology, Sheffield Teaching Hospitals, Sheffield, UNITED KINGDOM; 6 Adult Ophthalmology, Sheffield Teaching Hospitals, Sheffield, UNITED KINGDOM

**Introduction:** Behçet's disease (BD) is an idiopathic, multi system, auto-inflammatory vasculopathic disorder, characterised by mouth and genital ulcers, rash, arthritis and uveitis. Ocular manifestations occur in up to 80% of patients and are associated with 30% risk of blindness. We present a challenging case of an adolescent with paediatric onset BD and aggressive refractory uveitis mainly affecting the left eye.

**Case description:** An 11 year old girl presented acutely with a three day history of a painful red left eye and blurred vision. Examination revealed bilateral uveitis with optic disc swelling, cystoid macular oedema (worse in left eye), 2 plus cells in her right eye, 3 plus cells in her left eye (according to standardisation of uveitis nomenclature classification) with snowballs and reduced visual acuity. A two-year history of mouth and genital ulcers was given and at presentation mild arthritis was detected. Initial treatment with intravenous methylprednisolone (IVMP), intensive topical steroid eye drops and subcutaneously administered methotrexate (15mg/m^2^) was commenced. Infliximab (6 mg/Kg intravenously four-weekly) and mycophenolate mofetil (MMF, 600mg/m^2^ orally twice-daily) were subsequently added for multi-focal chorioretinitis in the left temporal retina. Infliximab was changed to tocilizumab (8mg/Kg intravenously 2-weekly) in response to reduced vision as a result of persisting chronic cystoid macular oedema. Subsequently, tocilizumab was changed to adalimumab (40mg subcutaneously fortnightly), for repeated sight-threatening uveitis flare-ups. Fifteen months after initial presentation MMF and adalimumab were substituted for pulsed intravenous cyclophosphamide (initially 500mg/m^2^ fortnightly, increasing to a maximum 1g/m^2^ 3-weekly; total 6 doses) for pseudo-hypopyon, recurrent widespread multi-focal chorioretinitis and acute sight loss (6/60). Multiple pulses of IVMP and oral prednisolone were given throughout the treatment course in response to repeated flare-ups of inflammation associated with reduced visual acuity due to macular oedema. Despite cyclophosphamide treatment, further severe flare-ups of eye disease complicated by pseudo-hypopyon, optic-disc swelling, multi-focal choroiditis and pseudo-necrotising retinitis occurred. Following cyclophosphamide, interferon-alfa-2A (roferon A, 3 million units subcutaneously daily) was commenced resulting in sustained and complete remission of eye inflammatory features including previously refractory cystic macular oedema; vision improved significantly to 6/9+. Interferon-alfa-2A had been well tolerated and demonstrated clinical effectiveness from the first week. However, following further flares, the dose was increased to 6 million units subcutaneously daily. Ocular disease remained relatively stable for 6 months although the other BD manifestations, such as, mouth ulcers remained problematic. An acute episode occurred consisting of pseudo-hypopyon, panuveitis, multifocal choroiditis and exudative retinal detachment. During the course of the illness, two years and five months after presentation, the patient developed functional visual loss and was managed with psychology intervention. Eye inflammation improved significantly with pulsed IVMP, and continued to require IVMP. However, disease flares with recurrent skin rashes occurred. Recommendations were followed, from liaising closely with National Paediatric Behçet's Centre at Alder Hey Hospital. Methotrexate was discontinued, interferon-alfa-2A was discontinued and adalimumab 40mg started by weekly injection alongside azathioprine 50mg orally once daily, gradually increased to 125mg. Mouth and genital ulcers of BD have subsided although clinically the patient is now left with visual impairment in the left eye due to probable occlusive vasculitis related optic neuropathy. A well-planned and coordinated transition pathway is in process for this now adolescent patient. This involves a collaborative and iterative approach with both the adult uveitis team and the patient and family, to ensure that the psychological manifestations of this complex disease are addressed and well supported throughout the transition period and beyond. Throughout the treatment pathway we collaborated with colleagues in national centres of expertise for paediatric BD and uveitis.

**Discussion:** For this patient, it has been proven to be significantly challenging to achieve long lasting remission of the ocular manifestations in BD. However, the disease has always responded well to prompt interventions with pulsed IVMP.

**Key Learning Points**: The challenges of treating Behçet's uveitis to achieve remission whilst preserving vision are well recognised. This case adds to the emerging evidence for the use of interferon-alfa-2A in treating Behçet's uveitis. The psychological impact of both the chronic diseases and treatment burden must be routinely assessed in the management of young people. A robust and well planned transition pathway is essential for adolescents with chronic complex disease processes such as Behçet's uveitis.

**Disclosure: G. Cooper:** None. **J. Choi:** None. **A. McMahon:** None. **C. Nash:** None. **L. Dunkley:** None. **R. Tattersall:** None. **F. Quhill:** None. **H. Lee:** None. **R. Verstegen:** None. **D. Hawley:** None.

